# STAT3 activation by catalpol promotes osteogenesis-angiogenesis coupling, thus accelerating osteoporotic bone repair

**DOI:** 10.1186/s13287-021-02178-z

**Published:** 2021-02-04

**Authors:** Liang Chen, Ri-Yan Zhang, Jun Xie, Jia-Yi Yang, Kang-Hao Fang, Chen-Xuan Hong, Rong-Bo Yang, Najeeb Bsoul, Lei Yang

**Affiliations:** 1 Department of Orthopaedic Surgery, The Second Affiliated Hospital and Yuying Children’s Hospital of Wenzhou Medical University, Wenzhou, 325000 China; 2 Key Laboratory of Orthopaedics of Zhejiang Province, Wenzhou, 325000 China; 3 School of Ophthalmology and Optometry, Wenzhou Medical University, Wenzhou, 325000 China; 4 Department of Gynecology, The First Affiliated Hospital of Wenzhou Medical University, Wenzhou, 325027 China; 5 Medical College, Zhejiang Jiaxing College, Jiaxing, 314000 China

**Keywords:** Catalpol, Bone marrow mesenchymal stem cells, JAK2/STAT3 signaling, Osteoporosis, Bone repair

## Abstract

**Background:**

Bone fracture repair has gained a lot of attention due to the high incidence of delayed union or even nonunion especially in osteoporotic patients, resulting in a dreadful impact on the quality of life. However, current therapies involve the costly expense and hence become unaffordable strategies for fracture recovery. Herein, developing new strategies for better bone repair is essential and urgent. Catalpol treatment has been reported to attenuate bone loss and promote bone formation. However, the mechanisms underlying its effects remain unraveled.

**Methods:**

Rat bone marrow mesenchymal stem cells (BMSCs) were isolated from rat femurs. BMSC osteogenic ability was assessed using ALP and ARS staining, immunofluorescence, and western blot analysis. BMSC-mediated angiogenic potentials were determined using the western blot analysis, ELISA testing, scratch wound assay, transwell migration assay, and tube formation assay. To investigate the molecular mechanism, the lentivirus transfection was used. Ovariectomized and sham-operated rats with calvaria defect were analyzed using micro-CT, H&E staining, Masson’s trichrome staining, microfil perfusion, sequential fluorescent labeling, and immunohistochemistry assessment after administrated with/without catalpol.

**Results:**

Our results manifested that catalpol enhanced BMSC osteoblastic differentiation and promoted BMSC-mediated angiogenesis in vitro. More importantly, this was conducted via the JAK2/STAT3 pathway, as knockdown of STAT3 partially abolished beneficial effects in BMSCs. Besides, catalpol administration facilitated bone regeneration as well as vessel formation in an OVX-induced osteoporosis calvarial defect rat model.

**Conclusions:**

The data above showed that catalpol could promote osteogenic ability of BMSC and BMSC-dependent angiogenesis through activation of the JAK2/STAT3 axis, suggesting it may be an ideal therapeutic agent for clinical medication of osteoporotic bone fracture.

## Background

Osteoporosis is becoming a worldwide health issue and a social threat due to the aging society. It is characterized by decreasing bone mass and deteriorating micro-architecture in the skeleton, both of which enhance the risk of bone fracture [1–3]. Even though the healing capacity of the bone tissue has been proved, a promising recovery of bone defect continues to be a challenging issue [4]. Various medical applications including autologous bone grafts and autografts have been used in the clinical options. However, these techniques encounter limitations because of the donor unavailability and morbidity, infection, immune rejection, pain, and the cost [5]. Herein, other therapeutic alternatives are necessary.

It is believed that bone formation, as well as fracture healing, is associated with moderate osteogenesis and angiogenesis [6]. Meanwhile, the communication between the BMSCs and vascular endothelial cells seems to be vital in the process of bone remodeling, which can be augmented by multiple regulatory factors that incorporate in the signaling modes of autocrine and/or paracrine, so as to participate in the recruitment, proliferation, and differentiation of BMSCs and vascular endothelial cells [7]. This connection has been demonstrated to have resulted from the osteoblastic and angiogenic factor production such as VEGF [8]. However, osteogenesis and angiogenesis under osteoporosis conditions are normally impaired due to the decreased recruitment of the osteoblastic cells to the damaged areas, consequently resulting in a nonunion of bone fracture [9–12]. Therefore, maintaining osteogenesis and angiogenesis is crucial for the osteoporotic bone regeneration.

Various signaling pathways are reported to be involved in the process of bone regeneration, among which Janus kinase/signal transducers and activators of transcription (JAK/STAT) pathways are indispensable. STAT3 is activated and phosphorylated by the JAK family particularly JAK2. This leads to STAT3 dimerization and nucleus translocation, and the initiation of the subsequent biological responses [13]. Previous studies have reported that the increase of the phosphorylation-STAT3 in BMSCs promotes osteogenic differentiation and enhances bone defect healing [14, 15]. Similarly, specific knockout of STAT3 potently reduces bone mineral density in mice [16]. In addition, JAK2/STAT3 axis inhibition contributes to the suppression of VEGF expression in bone marrow multipotent progenitor cells and BMSCs as well [17, 18]. These provide evidence that osteogenesis and angiogenesis in bone healing are greatly associated with the activation of the JAK2/STAT3 pathway.

Rehmanniae is a traditional Chinese herbal medicine that is used for the treatment of osteoporosis for hundreds of years [19]. However, its active part as well as the mechanism underlying its function has not been exhaustively explored. Catalpol is a natural iridoid glycoside that is dominantly enriched in the dried root of Rehmanniae, which possesses a diverse range of biological activities, such as anti-ischemic, anti-oxidative, and anti-inflammatory effects [20–22]. However, its potential actions on bone formation have not been fully elucidated. Recently, the association between the JAK2/STAT3 pathway and the actions of catalpol has been previously reported [23].

On this basis, we hypothesized that catalpol could promote BMSC osteogenesis and BMSC-mediated angiogenesis by activating the JAK2/STAT3 axis. The current study was to lay the foundation for the application of catalpol in the treatment of the clinical osteoporotic bone fracture.

## Methods

### Reagents and antibodies

Catalpol (> 96%) was purchased from Sigma-Aldrich (St. Louis, MO, USA). Primary antibodies against alpha-1 type I collagen (COL1A1), runt-related transcription factor 2 (RUNX2), and osteocalcin (OCN) were from Abcam (Cambridge, UK). The primary antibody against VEGF, CD31, p-JAK2, t-JAK2, p-STAT3, t-STAT3, and β-actin was obtained from the Cell Signaling Technology (Danver, MA, USA). Fetal bovine serum (FBS), minimum essential medium-alpha modification (α-MEM), and penicillin/streptomycin were purchased from Gibco BRL (Thermo Fisher Scientific; Waltham, MA, USA). All the other chemicals used were of analytical grade complying with the tissue and cell culture standards.

### Isolation and culture of rat BMSCs

BMSC isolation was performed as previously described [24]. Briefly, 2-week-old Sprague-Dawley rats were euthanized and sterilized in 75% ethanol for 15 min. BMSCs were flushed out by injection of modified Eagle’s medium alpha modification (α-MEM; HyClone, PA, USA) using a 5-ml syringe fitted with a 25-gauge needle under sterile conditions. After centrifugation, the BMSCs were cultured in α-MEM supplemented with 10% fetal bovine serum (FBS; Gibco, New York, USA) and 1% penicillin/streptomycin (Gibco) and incubated at 37 °C with 5% CO_2_. The BMSCs from passages 3–5 were used in all experiments.

### Cytotoxicity assay

The effect of catalpol on BMSC viability was determined by Cell Counting Kit-8 assay using commercial kits (MedChemExpress LLC; Monmouth Junction, NJ, The USA). Briefly, BMSCs were seeded in a 96-well plate at a density of 5 × 10^3^ cells per well and various concentrations of catalpol were added (0, 10, 25, 50, 100, 250, 500 μM) for 24 or 48 h. At the end of the experiment, a 10-μl CCK-8 solution was added into the wells. The cells were cultured for 2 more hours. The optical density (OD) at the wavelength of 450 nm was measured via a Multiskan Go Microplate Spectrophotometer (Thermo Fisher Scientific).

### Osteoblastic determination and mineralization protocols

Initially, BMSCs were seeded at a density of 5 × 10^4^ cells per well in a 24-well plate. After reaching over 80% confluence, the medium was replaced with osteogenic medium (complete α-MEM containing 1 nM dexamethasone, 50 μM ascorbic acid, and 20 mM β-glycerophosphate). For treatment, catalpol (50, 100, and 250 μM) was added into the medium and the untreated wells were redeemed as the control group. Total cellular proteins were extracted after 7 days of osteogenic induction for western blot analysis of proteins involved in osteoblastic differentiation. Alkaline phosphatase (ALP) activity was detected on the 7th day of differentiation using the ALP Staining Kit (Beyotime Institute of Biotechnology; Jiangsu, China). The mineralization of calcium nodule was determined at the 21st day using Alizarin Red S (ARS) solution (Solarbio Science & Technology). The absorbance at 520 nm for ALP and 570 nm for ARS staining was detected using a microplate reader.

### Western blotting

The total cell protein was extracted from the cultured cells using RIPA lysis buffer (Beyotime Institute of Biotechnology), containing protease and phosphatase inhibitors (Sigma-Aldrich), for 30 min at 4 °C. The cell lysates were cleared by centrifugation, and the protein concentration was determined using the BCA Protein Assay Kit (Beyotime Institute of Biotechnology) with respect to the manufacturer’s instruction. For each sample, 20 μg of the extracted protein (diluted in SDS Sampling buffer and denatured by boiling for 5 min) were resolved on a 10–15% SDS–polyacrylamide gel electrophoresis gel. The separated proteins were then transferred to polyvinylidene difluoride (PVDF) membranes (Merck Millipore; Burlington, MA, USA) overnight at 4 °C. PVDF membranes were blocked with 5% skim milk diluted in Tris-buffered saline with a 0.1% Tween 20 (TBST) for 2 h at room temperature. They were then incubated with the primary antibodies for 12 h at 4 °C. After extensive washes with TBST, the membranes were incubated with the corresponding HRP-conjugated secondary antibodies for 4 h at room temperature. The proteins were visualized through chemiluminescence and imaged on a ChemiDoc XRS+ (Bio-Rad; Hercules, CA, USA). The protein bands were quantified by densitometry analysis using Image Lab V3.0 software (Bio-Rad).

### Determination of angiogenesis-related functions of BMSCs

To determine whether catalpol could enhance BMSC-mediated angiogenic ability, the cells received 100 μM catalpol treatment and they were cultured for 0, 12, 24, or 48 h. Besides, BMSCs were treated with various concentrations of catalpol (0, 50, and 100 μM) for 24 h. The protein was extracted and the supernatant liquid harvested at the end of the treatment, for western blotting analysis and ELISA test of VEGF. For further assessment of the angiogenic capability, the BMSCs were incubated with/without 100 μM catalpol for 24 h, and the conditioned mediums were harvested and centrifuged at 2000×*g* for 10 min to obtain the supernatants, which were used for downstream experiments. Subsequently, HUVECs were cultured under different treating conditions: (1) fresh medium, (2) fresh medium added with 100 μM catalpol, (3) conditioned medium from BMSCs without catalpol, and (4) conditioned medium from BMSCs with 100 μM catalpol.

### ELISA testing

Commercial ELISA kit for VEGF (Cusabio, Wuhan, China) was used to determine the concentrations of VEGF in the culture medium after BMSC administration indicated the treatment, in accordance with the manufacturer’s protocols.

### Migration assays

Human umbilical vein endothelial cells (HUVECs) were purchased from Procell Life Science &Technology Company (Wuhan, China). For the scratch wound assay, HUVECs were seeded in a 12-well plate at a density of 2.0 × 10^5^/well. After confluence, the cells were scratched using a sterile pipette tip under an inverted microscope (Nikon; Tokyo, Japan). The cells were then cultured in mediums aforementioned. The wound images were obtained immediately, 12 and 24 h later. The width of the wounded areas was calculated as previously described: width of the area (%) = (A_0_–A_n_)/A_0_ × 100, where A_0_ represents the initial wound area, and A_n_ represents the residual wound area at the metering point. For the transwell migration assay, HUVECs were suspended at a density of 1 × 10^4^/well and loaded into the top chamber of a 24-well, 8-μm pore-size transwell plate (Corning, NY, USA). Then, the medium from the treated BMSCs was added to the below chamber. After 10 h, the un-migrated HUVECs in the upper chambers were removed by wiping the top of the membranes with cuspidal cotton swabs. After fixing in a 4% paraformaldehyde and washing with PBS solution, the migrated cells which were stained with 0.5% crystal violet for 2 h. They were imaged as well as counted under the random fields of the microscope (Nikon; Tokyo, Japan).

### Tube formation assay

HUVECs were seeded at the density of 1.5 × 10^4^/well onto Matrigel-coated 96-well plate and then incubated in the medium aforementioned. After 8 h of culture, the tube formation of HUVECs was observed under an inverted microscope (Nikon; Tokyo, Japan). The number of tubes was determined using the Image-Pro Plus software.

### Immunofluorescence

After the treatment, the cells were fixed in a 4% paraformaldehyde for 10 min at room temperature and permeabilized with a 0.5% (v/v) Triton X-100 in PBS for 20 min. Non-specific antibody binding was blocked with a 1% (w/v) goat serum albumin for 1 h at room temperature. It was then incubated with indicated antibodies (the dilution for the antibodies was 1:200 respectively) in a 0.2% BSA-PBS overnight at 4 °C, with gentle mechanical rocking. The cells were washed extensively and incubated with fluorescence conjugated secondary antibodies (Alexa Fluor 488 or 594; Thermo Fisher Scientific) for 1 h at room temperature, in the dark. The nuclei were stained with DAPI for 5 min at room temperature. Fluorescence images were captured on an Olympus BX53 fluorescence microscope (Olympus Life Science; Tokyo, Japan). The level of expression was determined using integrated optical density using the Image-Pro Plus software (Media Cybernetics, Inc.; Rockville, MD, USA).

### Lentivirus infection

STAT3 was suppressed by STAT3 knockdown lentivirus transfection (GeneChem, Shanghai, China). The BMSCs received STAT3 knockdown lentivirus or vehicle lentivirus transfection. After 12-h incubation, the medium was replaced and the cells were cultured for 2 days to reach a 90–95% confluence. The transfected cells were used in the subsequent experiments. The successfully transfected cells were observed under a fluorescence microscope (Olympus Life Science; Tokyo, Japan) whereas the untreated cells were invisible. The impact of STAT-3 knockdown transfection was detected via western blot analysis.

### Surgery and treatment

All the animal experiments were approved by the Animal Ethics Committee of The Second Affiliated Hospital and the Yuying Children’s Hospital of Wenzhou Medical University. They were carried out with respect to the criteria outlined in the Guide for the Care and Use of Laboratory Animals (NIH, Bethesda, MD, USA). Thirty 3-month-old SD female rats were purchased from the Shanghai Laboratory Animal Center (SLACCAS; Shanghai, China). They were housed in ventilated cages in groups of 5 under the SPF conditions of 22–25 °C and 12-h daylight. Twenty of the 30 rats were subjected to bilateral ovariectomy, and the rest underwent the sham operation and regarded as the SHAM group. After 3 months, the rats were screened to prove osteoporotic bone loss through the X-ray radiograph imaging. Then, all the rats were anesthetized, and 4-mm calvarial bone defects were made to determine osteogenesis and angiogenesis function as described in our previous study [25, 26]. After surgery, all the ovariectomized (OVX) rats were divided into 2 groups: OVX and OVX + catalpol. The rats in the OVX + catalpol group received 10 mg/kg/day catalpol intraperitoneal injection while those in the other groups were subjected to vehicle (PBS) treatment. The treatments were performed daily for 8 weeks. At the end of the treatment, all rats were euthanatized and the calvarial bones were harvested and fixed in a 4% formaldehyde.

### Micro-CT

Microstructural analysis of the calvaria of the rats was scanned on a micro-CT system and the associated software (μCT 100, Scanco Medical; Brüttisellen, Switzerland). The images were acquired at a voltage of 70 kV, an electric current of 200 μA, and a spatial resolution of 14.8 mm in all the directions. Three-dimensional reconstructed images were generated. The volume of interest (VOI) analyzed included the trabecular compartment 2 mm below the highest point of the growth plate to distal 100 CT slices. The volume of the regenerated bone within the calvaria defects, and bone formation was analyzed using the built-in software.

### Microfil perfusion

Calvarial blood vessel formation in the defect area was evaluated by perfusion with Microfil compound (Microfifil MV-122; Flow Tech) 8 weeks after craniotomy surgery. The rat was initially scraped clean and immobilized on a foam board following anesthesia. An incision was created between the xiphoid and the abdomen. The inferior vena cava was cut and flushed with PBS containing sodium heparin (100 U/ml). The vessel was then fixed via injection of 4% formaldehyde. Then, 20 ml of silicone rubber compound was injected into the vessel at a rate of 2 ml/min. Later, the specimen was stored overnight at 4 °C, and fixed in a 4% paraformaldehyde for a further 48 h. The tissues were then after decalcified in 10% ethylene diamine tetra acetic acid (EDTA; SigmaAldrich, MO) for 4 weeks. Finally, the vessel formation of the cranial defect was analyzed using a micro-CT system.

### Sequential fluorescent labeling

Continuous fluorescent labeling for the detection of bone mineralization in calvaria defects was carried out. Rats were injected with calcein (15 mg/kg of body weight, CA Sigma USA) intraperitoneally at 4 and 6 weeks after the surgery. Fluorescent agents were prepared instantly before injection and filtered using a 0.45-μm filter. The calvaria tissue samples were harvested for hard-tissue slicing and imaged through the fluorescence microscopy (Olympus Life Science; Tokyo, Japan).

### Histology and immunohistochemistry assessment

Fixed calvaria bone tissues were decalcified in 10% EDTA for 3 weeks for histological and immunohistochemical (IHC) analyses. Then, the specimen was cut in half, dehydrated in graded ethanol series (70% to 100%), cleared in xylene, and paraffin-embedded for sectioning. Four-micrometer-thick sections were then subjected to hematoxylin and eosin (H&E), and Masson’s trichrome staining as per the standard laboratory protocols. For IHC staining, 6-μm sections were incubated with primary antibodies against CD31, VEGF, and OCN. The immune-reactivities of the sections were determined using horseradish peroxidase detection system in accordance with manufacturer’s protocols (Vector Laboratories; Burlingame, CA, USA).

### Statistical analyses

All statistical analyses were carried out using the GraphPad Prism software (San Diego, CA, USA). The data presented herein are expressed as the mean ± standard error of the mean from at least three experimental repeats. Two-tailed Student’s *t* test was used to compare the means between two groups and one-way ANOVA with Bonferroni or Dunnett corrections were used for multiple comparisons where appropriate. Differences were determined to be statistically significant when *p* < 0.05.

## Results

### Catalpol enhanced osteogenesis of BMSCs in vitro

**Fig. 1:**
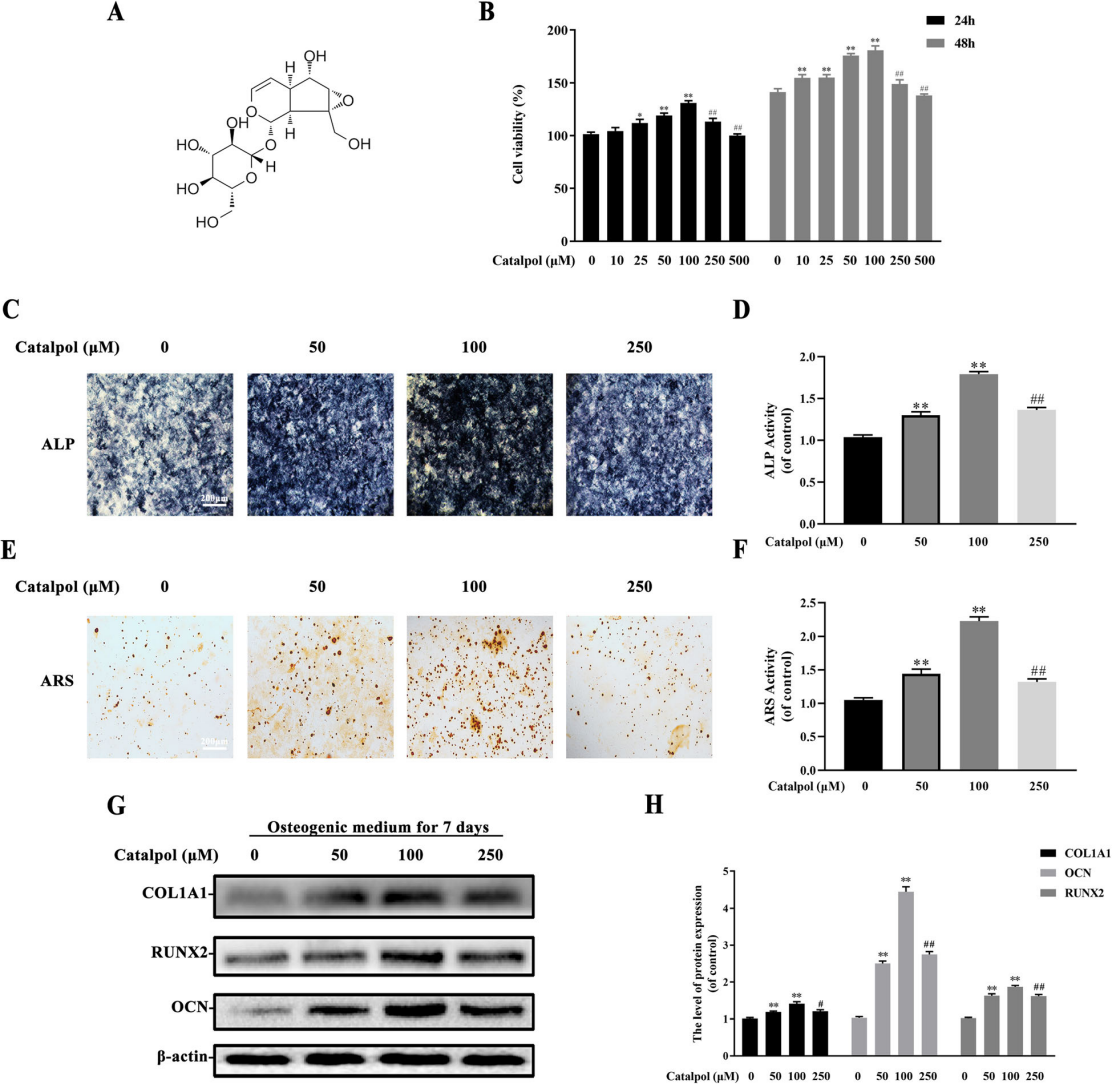
Catalpol enhanced osteogenesis of BMSCs in vitro. **a** Catalpol chemical structure. **b** The effect of catalpol on BMSC proliferation was measured per CCK-8 assays. **c**, **d** Images and quantification of ALP activity after 7 days of osteogenic induction. **e**, **f** Calcium mineralization was assessed via ARS staining and quantification. **g**, **h** Western blot analysis of osteogenic-related proteins COL1A1, RUNX2, and OCN. All the experiments were repeated at least 3 times independently. The data are presented as means ± SEM. ^*^p < 0.05, ^**^p < 0.01 vs. control group, ^##^*p* < 0.01 vs. 100 μM catalpol group

Catalpol is a natural iridoid glycoside. It is a monoterpene containing a glucose molecule (Fig. 1a). To determine whether catalpol affects the proliferation of BMSCs, we initially performed CCK-8 assays. Cell proliferation was not significantly suppressed by treatment with 10, 25, and 50, 100 μM catalpol for 24 h or 48 h compared to the control group (Fig. 1b). However, there was no more favorable change in cell viability when incubated with higher concentrations of catalpol (250, 500 μM, *P* < 0.05). Then, we assessed the pro-osteogenic effect of catalpol on BMSCs in vitro. After 7 days of osteoblastic induction, BMSCs treated with 50 or 100 μM catalpol indicated an increasing level of ALP activities (Fig. 1c, d), in comparison to the control (*P* < 0.05). Consisting with the result of CCK-8 assay, the rewarding effect was partly attenuated in BMSCs when incubated with a higher concentration of catalpol (250 μM). It was also validated by ARS staining, which epresented extracellular deposition of calcium after 21 days of osteogenic induction (Fig. 1e, f). Accordingly, the results of the western blot analysis of osteogenesis-related proteins COL1A1, RUNX2, and OCN also revealed that catalpol promoted osteoblastic gene expression compared to the control (*P* < 0.05) (Fig. 1g, h).

### Catalpol augmented BMSC-dependent angiogenesis of HUVECs

**Fig. 2:**
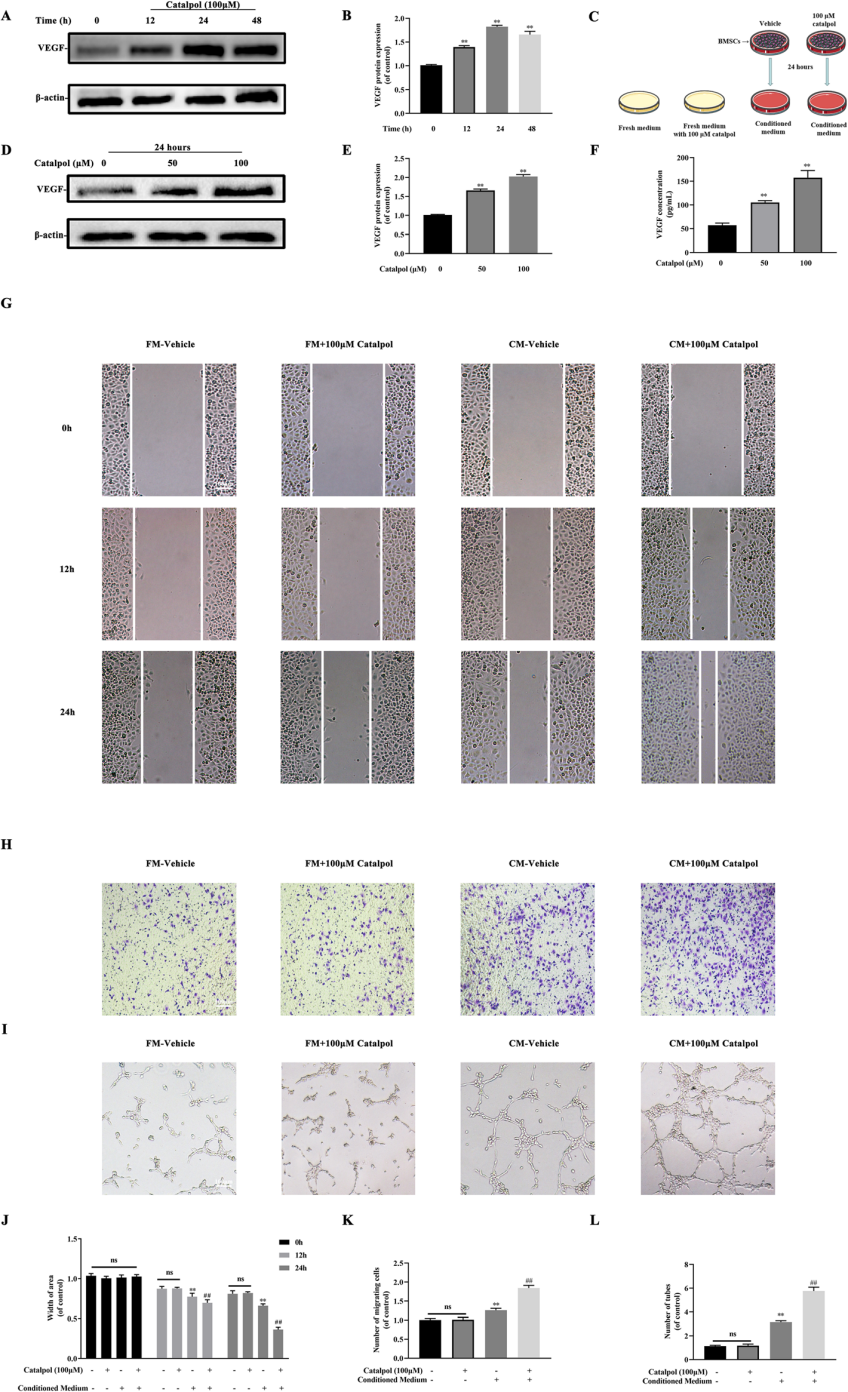
Catalpol augmented BMSC-dependent angiogenesis of HUVECs. **a**, **b** Western blot analysis of VEGF expression in BMSCs after indicated treatment. **c** Diagram of groups dividing and treatment respectively, **d**, **e** Western blot analysis of VEGF expression in BMSCs after indicated treatment. **f** VEGF secretion in the supernatant liquid from different groups was assessed using ELISA kits. Images and quantification of **g**, **j** scratch wound assay, **h**, **k** transwell migration assay, and **i**, **l** tube formation assay of HUVECs incubated with indicated mediums. The data are presented as means ± SEM. All experiments were repeated at least 3 times independently. ^**^p < 0.01 vs. control group, ^##^*p* < 0.01 vs. CM-vehicle group. (FM fresh medium, CM conditioned medium)

Since VEGF is a critical pro-angiogenesis factor in the crosstalk between BMSCs and endothelial cells, we first evaluated the impact of catalpol on BMSCs VEGF expression and secretion. It was realized that VEGF expression was highest 24 h after catalpol application (Fig. 2a, b). We also observed that VEGF expression and secretion were enhanced in a dose-dependent manner (Fig. 2d–f). To further explore the catalpol effect on BMSC-dependent angiogenesis, we treated BMSCs with/without 100 μM catalpol for 24 h then collected the respective conditioned mediums to find out whether catalpol treatment could facilitate BMSC-induced pro-angiogenic impacts of HUVECs (Fig. 2c). As evidenced by the scratch wound assay and transwell migration assay, the conditioned medium from catalpol treated BMSCs resulted in an incredible increase in HUVEC migration in comparison to the untreated group (Fig. 2g, h, j, k, *P* < 0.05). Also, catalpol treatment enhanced BMSC-related capillary tube-like structures on Matrigel (Fig. 2i, l). Besides, our results demonstrated that catalpol treatment was not able to facilitate HUVEC angiogenesis. These findings suggested that catalpol possessed the ability to promote BMSC-mediated angiogenesis by promoting VEGF expression.

### JAK2/STAT3 axis activation contributed to the impacts of catalpol on osteogenesis-angiogenesis coupling

**Fig. 3:**
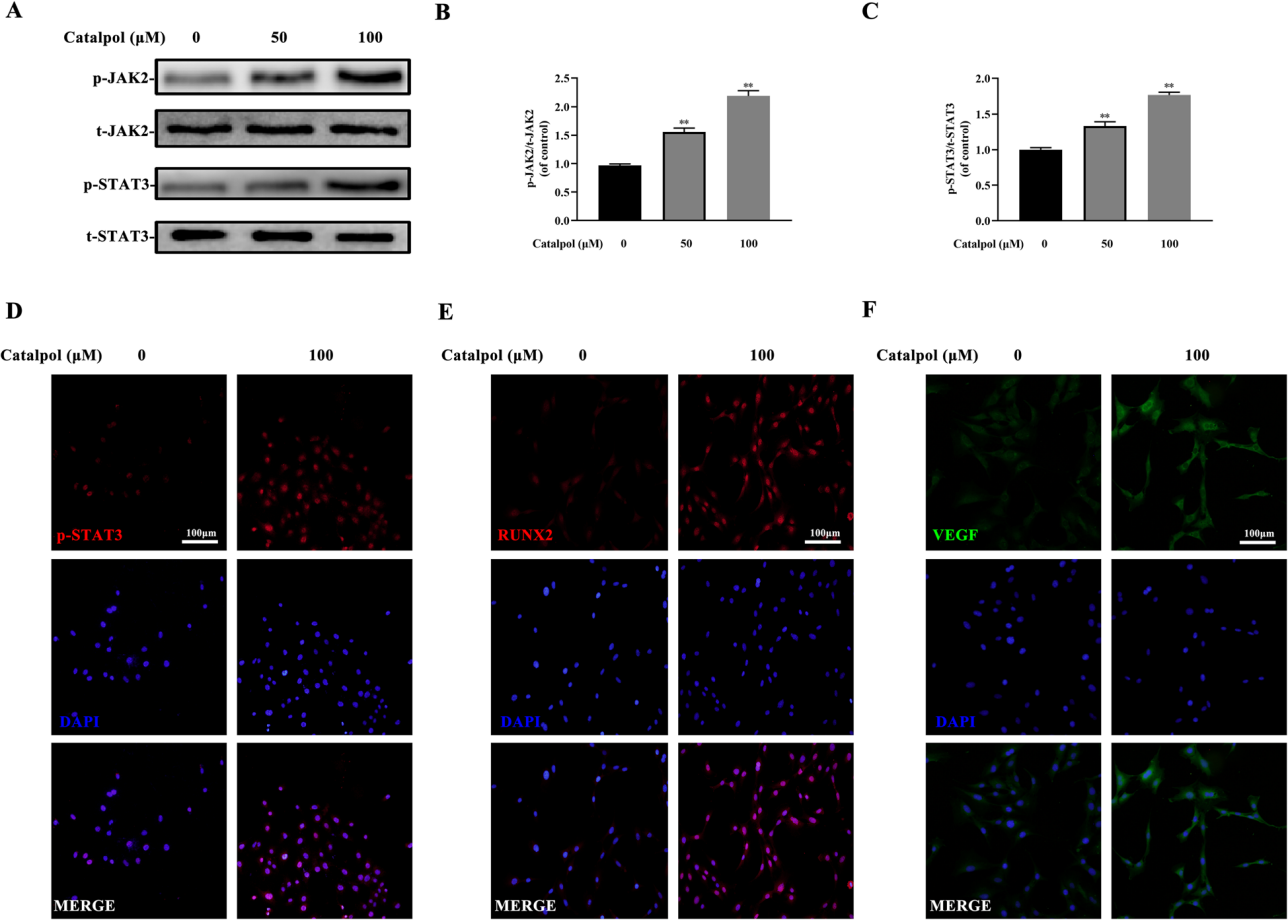
The effect of catalpol action was via the JAK2/STAT3 pathway. **a** Western blot results of t/p-JAK2 and t/p-STAT3 of different treated BMSCs. **b**, **c** Quantification of western blot results. Images of immunofluorescence of **d** p-STAT3, **e** RUNX2, and **f** VEGF in BMSCs. The data are presented as means ± SEM. All experiments were repeated at least 3 times independently. ^**^p < 0.01 vs. control group

**Fig. 4:**
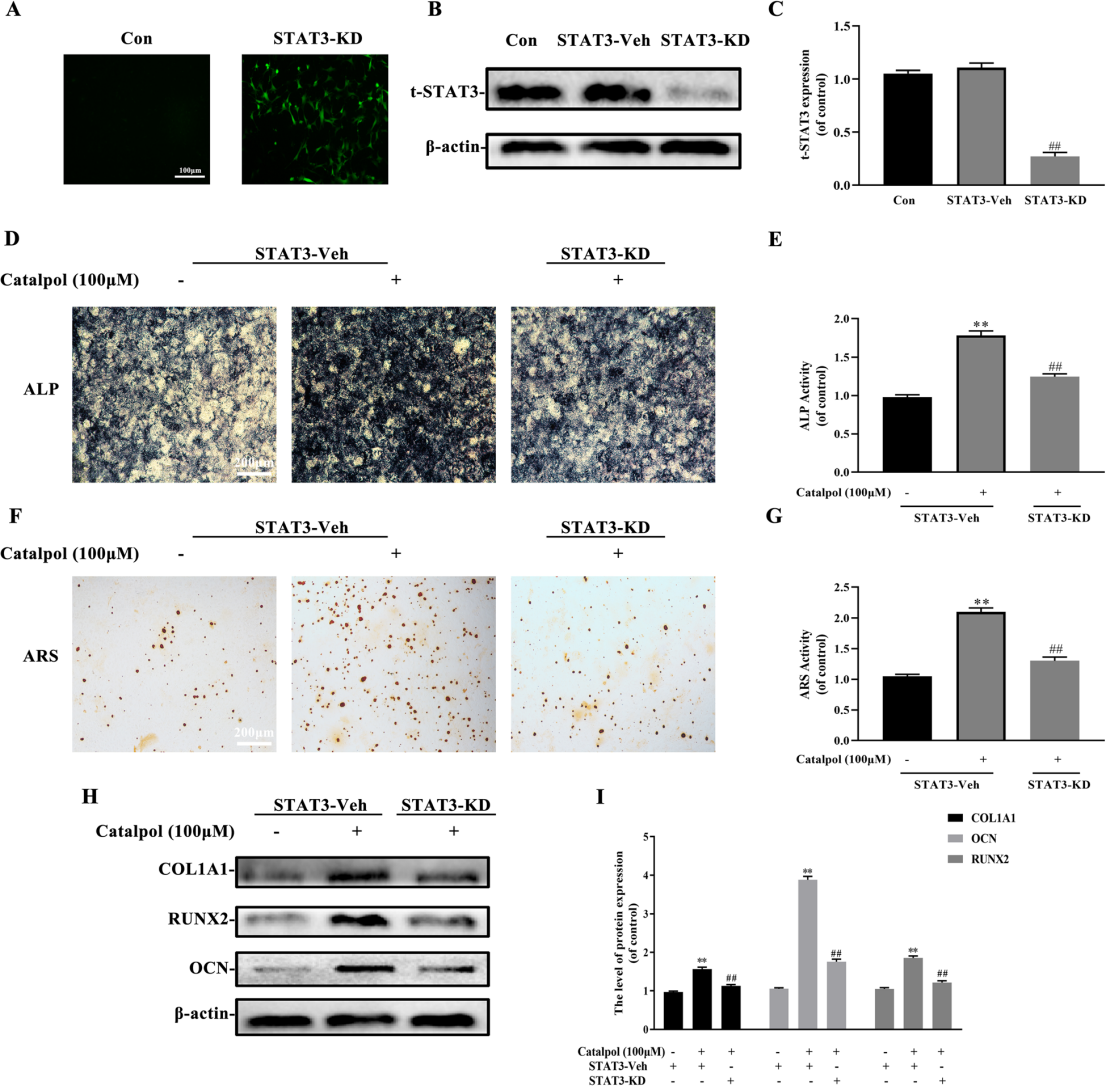
The knockdown of STAT3 partially reduced the effect of catalpol on BMSC osteogenesis. Successful transfection was demonstrated via **a** fluorescence microscope and **b**, **c** western blot analysis. BMSC osteogenic differentiation was evaluated by **d**, **e** ALP staining and **f**, **g** ARS staining. **h**, **i** western blot analysis of osteogenic-specific genes. The data are presented as means ± SEM. All experiments were repeated at least 3 times independently. ^**^p < 0.01 vs. control group, ^##^*p* < 0.01 vs. STAT3-vehicle group

**Fig. 5:**
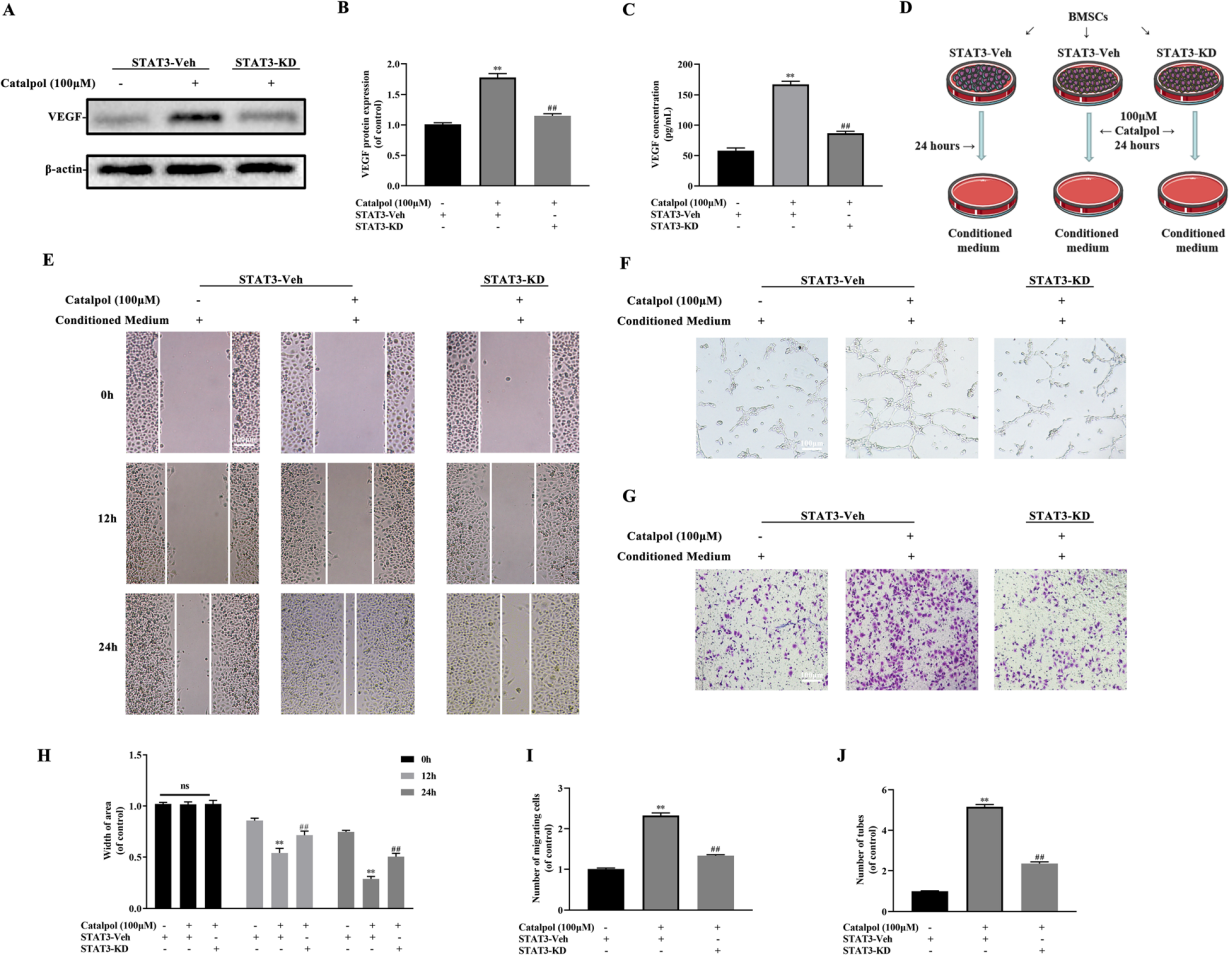
Angiogenic effects caused by catalpol treatment were partially abolished after STAT3-knockdown in BMSCs. VEGF expression and secretion of BMSCs was measured by **a**, **b** western blotting and **c** ELISA tests. **d** Diagram of groups dividing and treatment respectively, The pro-angiogenic ability of treated BMSCs was assessed via **e**, **h** scratch wound assay, **f**, **i** transwell migration assay, and (**g**, **j**) tube formation assay. The data are presented as means ± SEM. All experiments were repeated at least 3 times independently. ^**^p < 0.01 vs. control group, ^##^*p* < 0.01 vs. STAT3-vehicle group

To determine the role of JAK2/STAT3 pathway in the effect of catalpol, BMSCs were treated with 50 and 100 μM catalpol for 24 h. The western blot analysis result demonstrated that catalpol increased phosphorylation of JAK2 and STAT3 (Fig. 3a–c). Treatment of 100-μM catalpol significantly increased nucleus p-STAT3 translocation and the expression of RUNX2 and VEGF as proved by immunofluorescence (Fig. 3d–f). To further explore the role of STAT3 on the effect of catalpol, we suppressed STAT3 expression via lentivirus transfection. A successful transfection was carried out as the transfected BMSCs were fluorescent while the untreated cells were invisible under a fluorescent microscope (Fig. 4a). BMSCs treated with STAT3 knockdown lentivirus showed a lower level of STAT3 expression as compared to the others (*P* < 0.05) (Fig. 4b, c). We next determined if STAT3 was responsible for the effects of catalpol on BMSCs and realized that the knockdown of STAT3 diminished ALP activity and mineralization of BMSCs compared to the vehicle group (Fig. 4d–g). Parallel results showed that osteogenesis-related proteins RUNX2, COL1A1, and OCN were downregulated in STAT3 knockdown BMSCs (Fig. 4h, i). As shown in Fig. 5d, we next determined whether STAT3 was also involved in the pro-angiogenic effect of catalpol action. Our data showed that VEGF expression and secretion in catalpol-treated BMSCs were reduced after the STAT3 knockdown (Fig. 5a–c). A concomitant reduction in BMSC-induced pro-angiogenesis effect in the scratch wound assay, transwell migration assay, and tube formation assay was also observed (Fig. 5e–j). It was clear that this effect was attenuated after STAT3 blocking (*P* < 0.05). All these data demonstrated that the JAK2/STAT3 pathway was involved in the catalpol action on osteogenesis-angiogenesis coupling in vitro.

### Intraperitoneal administration of catalpol increased bone healing capacity in OVX calvarial defect rats

**Fig. 6:**
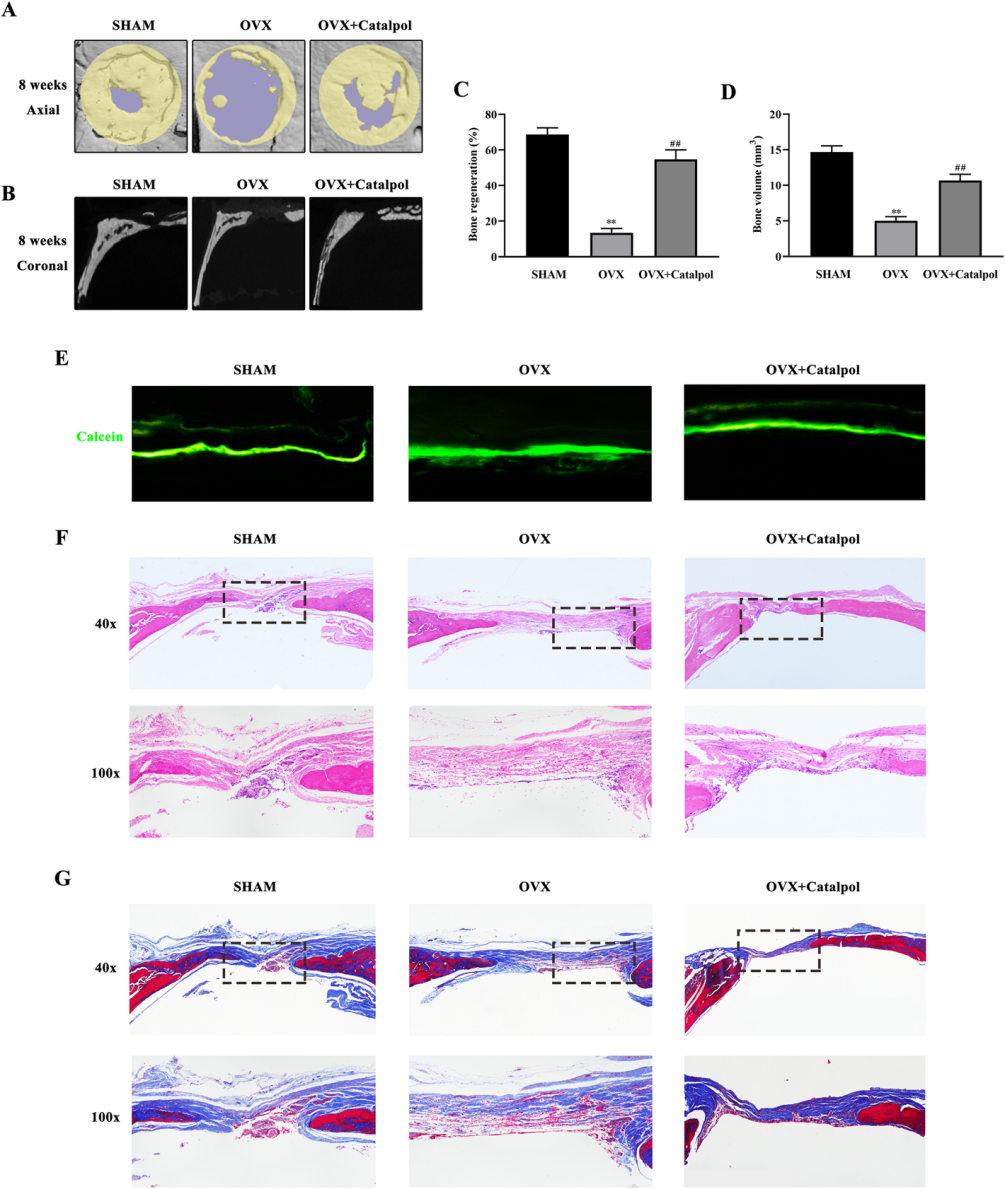
Intraperitoneal administration of catalpol increased bone healing capacity in OVX calvarial defect rats. **a–d** Micro-CT images and analysis of calvaria defect of different groups of rats. **e** New bone formation was detected by sequential fluorescent labeling of calcein. Histological assessment of the defect area by **f** H&E staining and **g** Masson trichrome staining. The data are presented as means ± SEM. ^**^p < 0.01 vs. SHAM group, ^##^*p* < 0.01 vs. OVX group

We explored if the effects of catalpol on BMSCs and HUVECs could be recapitulated in vivo. Rats were first ovariectomized and then subjected to craniotomy and received catalpol/vehicle treatment for 8 weeks. The 3D reconstruction images of calvaria showed that an increase in bone regeneration area in the OVX + catalpol group as compared to the OVX group (Fig. 6a–d). This confirmed that catalpol treatment promoted bone formation in OVX rats. Accordingly, bone mineralization labeled by calcein at 4 weeks and 6 weeks after craniotomy revealed that the distance strip in the OVX + catalpol group which marked by green was wider than in the OVX group (Fig. 6e). Meanwhile, the H&E staining and Masson trichrome staining of the calvarial bone showed that catalpol administration increased bone mineralization and formation around the defect than the OVX group (Fig. 6f, g).

### Catalpol treatment facilitated vessel formation in the defect area

**Fig. 7:**
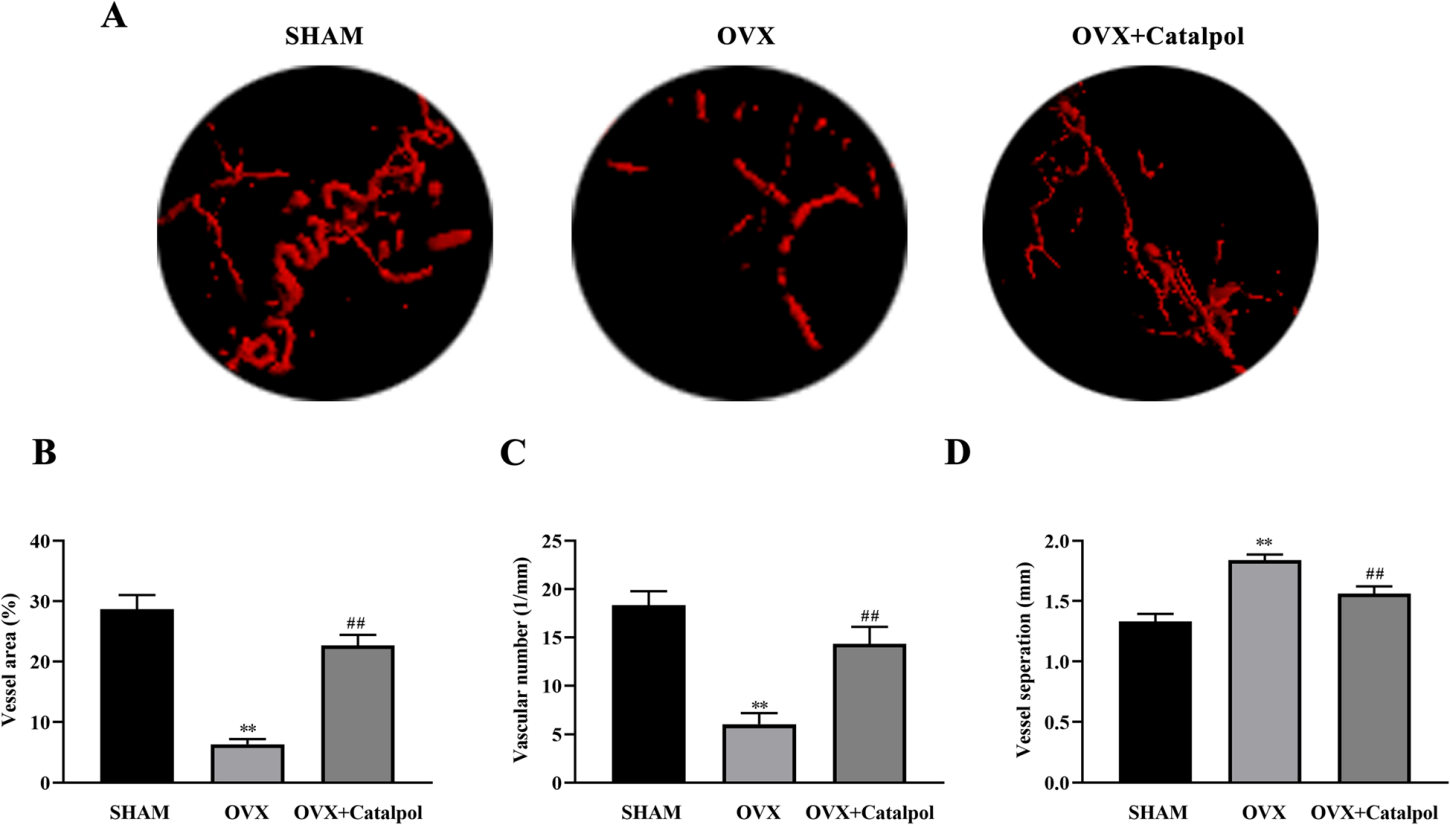
Catalpol treatment facilitated vessel formation in the defect area. **a** Images of Microfil perfusion within defect area. Analysis of **b** vessel area, **c** vascular numbers, and **d** vessel separation. The data are presented as means ± SEM. ^**^p < 0.01 vs. SHAM group, ^##^*p* < 0.01 vs. OVX group

To observe vascular formation in calvaria defect, the rats were perfused with Microfil. A sparse vessel network was found in the OVX group while more vascular formation was detected in the OVX + catalpol group (Fig. 7a). With regard to the quantitative analysis, both the vessel area and vascular number increased when treated with catalpol, concomitant with the decrease in vessel separation as compared to the OVX group (Fig. 7b–d). The results implied that catalpol treatment improved vessel formation in osteoporosis calvaria defect.

### Catalpol enhanced osteogenesis and angiogenesis in the calvarial defect in OVX rats

**Fig. 8:**
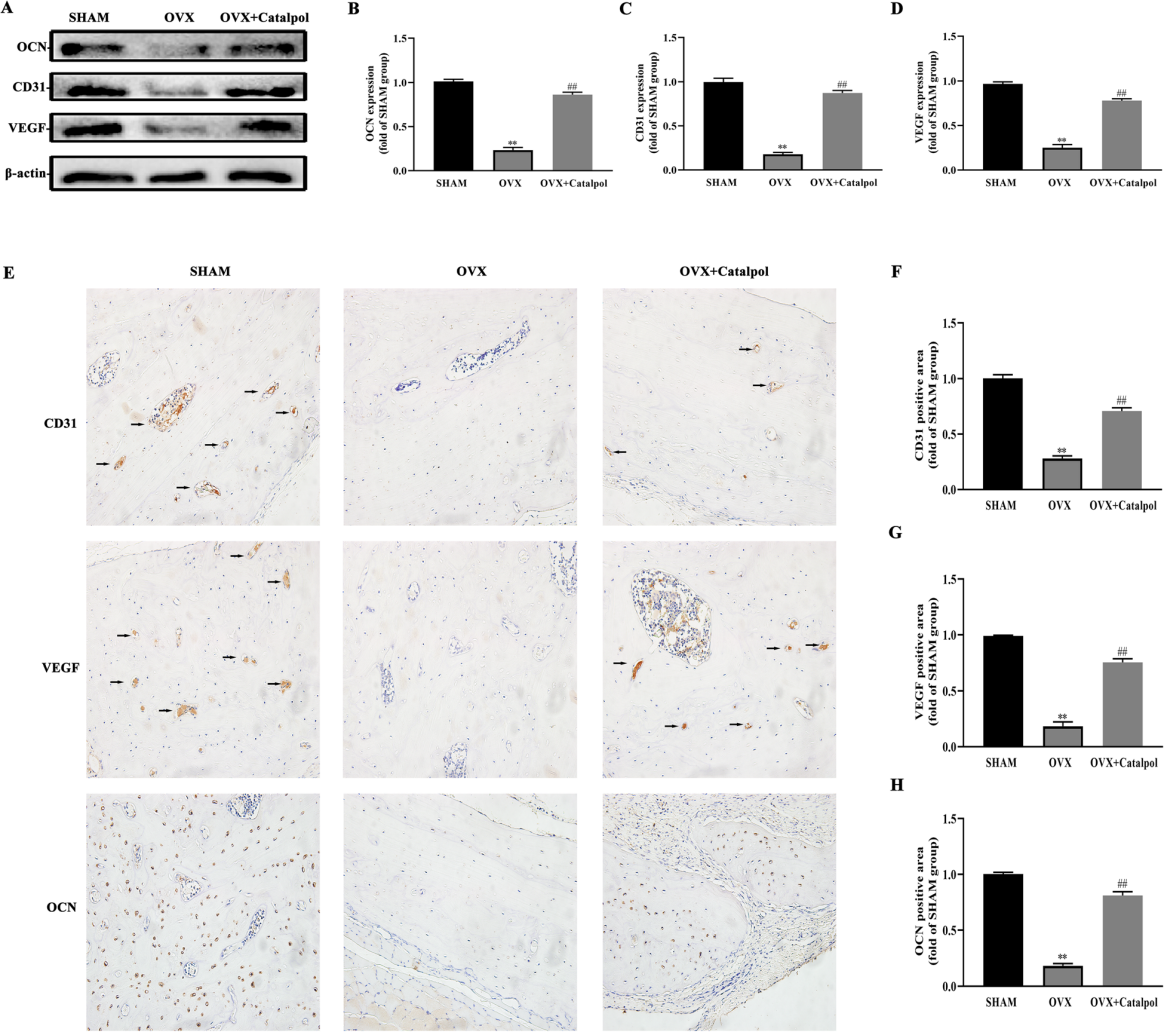
Catalpol enhanced osteogenesis and angiogenesis in calvarial defects in OVX rats. **a**–**d** Western blot analysis of OCN, CD31, and VEGF expression in defect area; **e**–**h** Images of immunohistochemical staining of CD31, VEGF, and OCN in the calvarial defect. The data are presented as means ± SEM. All experiments were repeated at least 3 times independently. ^**^p < 0.01 vs. SHAM group, ^##^*p* < 0.01 vs. OVX group

To evaluate osteogenesis and angiogenesis-related gene expressions, we extracted the proteins from the bone tissue surrounded the bone defect. Western blot results manifested that RUNX2, OCN, and VEGF expressions were upregulated in the OVX + catalpol group as compared to the OVX group (Fig. 8a–d). Besides, the immunohistochemical staining and quantitative analysis of OCN, VEGF, and vessel marker CD31 revealed a more positive area in the OVX + catalpol group (Fig. 8e–h). Generally, these findings revealed that catalpol treatment improved bone formation and neovascularization in OVX rats.

## Discussion

**Fig. 9:**
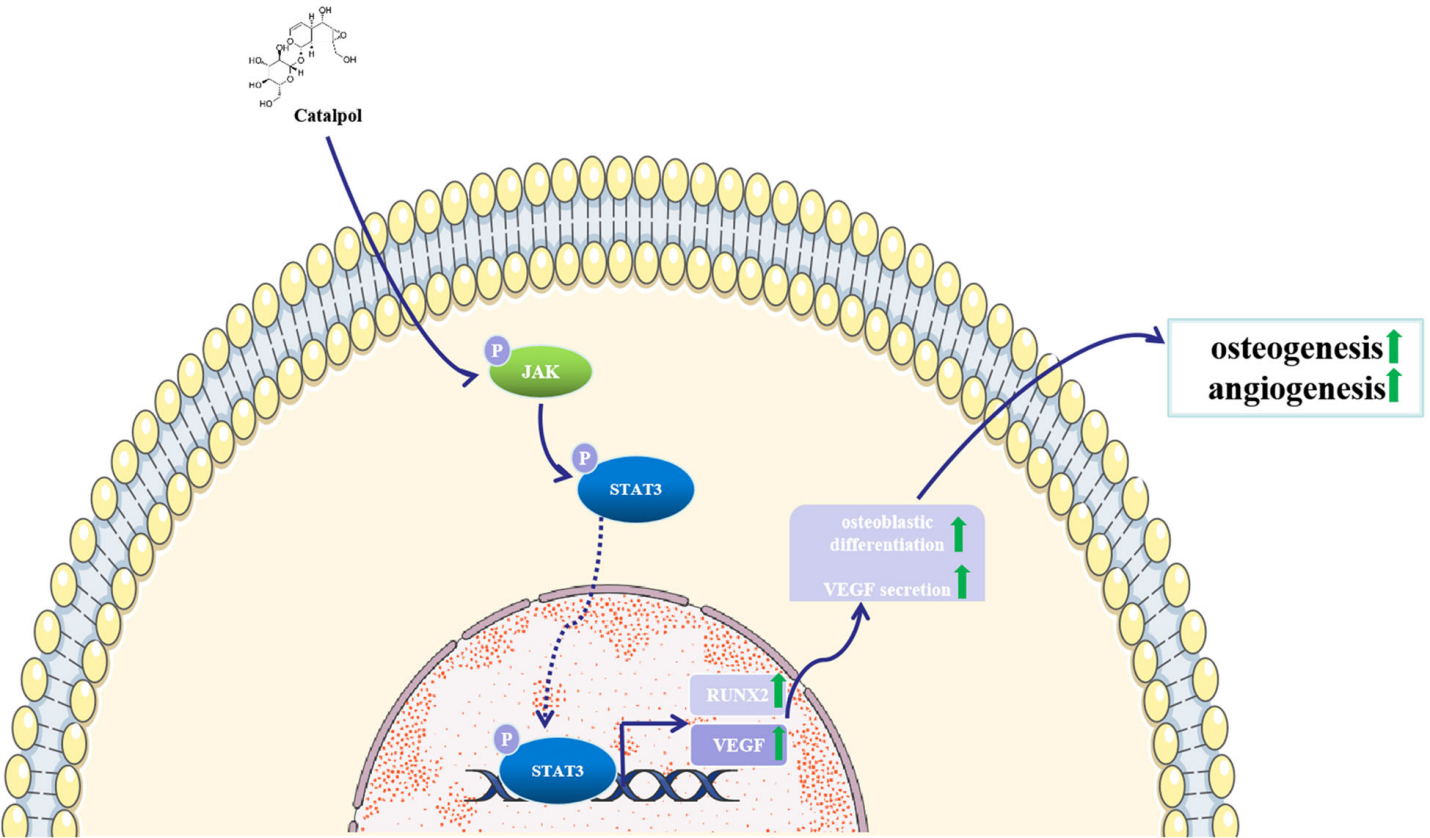
Mechanism of catalpol action on the effects in BMSCs

Bone defect repairing is a recapitulation process with complex signaling cascades, including spatiotemporal osteogenesis and angiogenesis, both of which require BMSC migration and osteogenic differentiation, accompanied by a well-organized blood vessel production [27, 28]. However, the delayed union or even nonunion of bone fracture often occurs in osteoporosis patients due to the decreased osteogenesis and disordered angiogenesis [29]. Therefore, exploring more therapeutic strategies and ideal drugs for the treatment of osteoporosis fracture is vital for clinical application. To the best of our knowledge, the current study is the first to demonstrate that catalpol treatment could enhance BMSC osteogenic determination and BMSC-mediated angiogenesis through the JAK2/STAT3 pathway activation. Further, knockdown of STAT3 partially abolished the beneficial effects of catalpol on BMSCs in vitro. Meanwhile, in vivo experiments confirmed that catalpol administration accelerated bone repair as well as promoted angiogenesis in an OVX rat calvaria defect model. The mechanism of catalpol actions on BMSCs was as displayed in Fig. 9.

BMSCs are identified as multipotent, undifferentiated cells with regenerative ability. The BMSCs play a central role in postnatal bone regeneration, which comprises two main ossification processes, namely, endochondral ossification and intramembranous ossification [30]. When a fracture happens, BMSCs spontaneously migrate to the bone lesion and directly differentiate to osteoblasts, which secrete extracellular matrix to help in rapid bone repair [27]. The improvement of BMSC osteogenic lineage function is essential for the successful fracture healing especially the nonunion. To this end, it is valuable to identify methods that could promote osteoblast differentiation of BMSCs for traditional medicine. To date, natural medicine has captured a lot of attention for its fascinating effects on promoting bone recovery. The study from Zhang et al. suggests that psoralen induced osteoblast determination and accelerated tibial fracture healing by activating ERK signaling [31]. Karvande et al. have reported the application of Heartwood extract from *Dalbergia sissoo* stimulates osteoblast function and promotes osteoporotic bone repair [32]. Rehmanniae, a traditional and official medicine which is listed in the Chinese Pharmacopeia, has been transformed into various therapeutic applications for multi-purpose clinical uses such as osteoporosis treatment [19, 33]. Recently, a growing number of researchers have reported that catalpol, the main component of Rehmanniae, possesses favorable pharmacological properties on bone metabolism. A study by Zhu et al. on the effect of catalpol on rat BMSCs showed that catalpol treatment could promote BMSC osteogenesis and prevent OVX-induced bone loss [21]. According to their results, the pro-osteogenic effect of catalpol was involved with activation of the Wnt/β-catenin pathway, as co-culture with Dickkopf-related protein 1 (DKK-1) partly inhibited the increased osteogenesis of BMSCs by treatment of catalpol. Their work was further validated by the study from Cheng et al., which demonstrated that catalpol could enhance the activity of Wnt/β-catenin axis in MC3T3-E1 osteoblasts under high glucose condition [34]. Choi et al. also reported that catalpol prevents MC3T3-E1 osteoblasts apoptosis caused by TCDD-induced damage [35]. Lai and his colleagues found that catalpol could alleviate bone loss by mediating the Th1/Th2 cells paradigm [36]. Consistent with the previous findings, we showed that catalpol treatment enhanced ALP activity of BMSCs and augmented calcium mineralization in vitro, accompanied with elevated levels of osteogenic gene expression. In vivo results also revealed that catalpol application contributed to bone defect repair in OVX rats, confirming that catalpol treatment was potentially helpful to bone tissue regeneration.

The coordinated coupling of osteogenesis and angiogenesis is vital in the maintenance of the bone mass and bone regeneration, as blood vessels carry oxygen, materials, and nutrients that are critical to the bone development [30]. The impairment of neovascularization could inhibit trabecular bone regeneration and retard fracture healing. For example, bone density and strength considerably decline after surgical damage to the blood vessels [9, 37]. Vascular endothelial growth factor (VEGF) has been regarded as one of the most critical factors for bone regeneration [38]. More importantly, VEGF from early osteoprogenitors is dispensable for periosteal angiogenesis and bone regeneration [12]. Our previous study also agreed with the perspective that vascular formation was indispensable in the bone repairing process [39, 40]. In this current study, our data proved that catalpol administration promoted vessel formation in the calvaria defect area of OVX rats. Besides, VEGF secreted from catalpol-treated BMSCs could induce a higher level of HUVEC angiogenesis in vitro, which is consistent with Ju et al.’s discovery.

Previous studies have depicted the roles of the JAK2/STAT3 signaling pathway in bone metabolism including osteogenesis and neovascularization [41]. The application of JAK2 inhibitor TG101348 as well as AG490 suppresses BMSC osteogenic differentiation and consequently impairs bone healing [42, 43]. Besides, STAT3 is the most influential transcription factor among the STATs family in mediating cellular signaling in bone-associated cells [44]. STAT3 regulates osteogenesis and angiogenesis through gene transcription by binding the specific gene-promoter sequences in the nucleus such as RUNX2 and VEGF [45, 46]. In humans, STAT3 mutation declines the bone mass and increases the incidence of trauma fractures [47]. This is normally the consequence of increased bone resorption and decreased bone formation. Besides, more evidence suggested that bone-selective STAT3 knockout mice indicated an osteoporotic phenotype [48, 49]. With regard to its role in vessel formation, the association between STAT3 and neovascularization has been widely elucidated. The activation of STAT3 promotes the angiogenic capability of endothelial cells both in vivo and in vitro [50]. Also, BMSC-secreted VEGF considerably decreased after the inhibition STAT3, resulting in a nonunion of the bone fracture [14, 18]. Work from Dong et al. demonstrated that the effect of catalpol was mediated by the JAK2/STAT3 pathway [23]. In this study, we observed that co-culture with catalpol triggered JAK2 as well as STAT3 phosphorylation and promoted STAT3 nucleus-translocation in the BMSCs. Results from our study also indicated that BMSC osteogenic determination and BMSC-secreted VEGF, as well as its concomitant angiogenic effects, were partly abrogated after STAT3 knockdown. Additionally, we found this catalpol effect on BMSC-dependent VEGF secretion was consistent with previous study from Ju and his colleagues [51]. In general, these results indicated that catalpol exerted a coupling effect between osteogenesis and angiogenesis in BMSCs through JAK2/STAT3 activation.

## Conclusions

Our findings highlight the beneficial effects of catalpol in promoting osteogenic and pro-angiogenic abilities of BMSCs. These impacts are preliminary considered via JAK2/STAT3 pathway activation. The application of catalpol may provide new insight and strategy for bone regeneration and hence could be an ideal therapeutic remedy against osteoporosis fracture.

## Data Availability

All data generated or analyzed during this study are included in this published article.

## Abbreviations

ALP: Alkaline phosphatase
ARS: Alizarin red S
BMSCs: Bone marrow mesenchymal stem cells
BCA: Bicinchoninic acid
BSA: Bovine serum albumin
COL1A1: Alpha-1 type I collagen
CCK-8: Cell counting Kit-8
DAPI: 4′,6-Diamidino-2-phenylindole
DKK-1: Dickkopf-related protein 1
ELISA: Enzyme-linked immunosorbent assay
EDTA: Ethylene diamine tetraacetic acid
FBS: Fetal bovine serum
H&E: Hematoxylin and eosin
HUVECs: Human umbilical vein endothelial cells
IHC: Immunohistochemical
JAK/STAT: Janus kinase/signal transducers and activators of transcription
OCN: Osteocalcin
PVDF: Polyvinylidene fluoride
PBS: Phosphate buffer saline
RUNX2: Runt-related transcription factor 2
SEM: Standard error of mean
TCDD: Tetrachlorodibenzo-p-dioxin
VOI: Volume of interest
α-MEM: Modified Eagle’s medium alpha modification
